# Predicting MHC-peptide binding affinity by differential boundary tree

**DOI:** 10.1093/bioinformatics/btab312

**Published:** 2021-07-12

**Authors:** Peiyuan Feng, Jianyang Zeng, Jianzhu Ma

**Affiliations:** Institute for Interdisciplinary Information Sciences, Tsinghua University, Beijing, China; Institute for Interdisciplinary Information Sciences, Tsinghua University, Beijing, China; MOE Key Laboratory of Bioinformatics, Tsinghua University, Beijing, China; Institute for Artificial Intelligence, Peking University, China

## Abstract

**Motivation:**

The prediction of the binding between peptides and major histocompatibility complex (MHC) molecules plays an important role in neoantigen identification. Although a large number of computational methods have been developed to address this problem, they produce high false-positive rates in practical applications, since in most cases, a single residue mutation may largely alter the binding affinity of a peptide binding to MHC which cannot be identified by conventional deep learning methods.

**Results:**

We developed a differential boundary tree-based model, named DBTpred, to address this problem. We demonstrated that DBTpred can accurately predict MHC class I binding affinity compared to the state-of-art deep learning methods. We also presented a parallel training algorithm to accelerate the training and inference process which enables DBTpred to be applied to large datasets. By investigating the statistical properties of differential boundary trees and the prediction paths to test samples, we revealed that DBTpred can provide an intuitive interpretation and possible hints in detecting important residue mutations that can largely influence binding affinity.

**Availability and implementation:**

The DBTpred package is implemented in Python and freely available at: https://github.com/fpy94/DBT.

**Supplementary information:**

Supplementary data are available at *Bioinformatics* online.

## 1 Introduction

Cytotoxic T lymphocytes destroy cancer or virus infected cells by expressing T-cell receptors (TCRs) that can recognize specific antigens. In most cases, an antigen is a linear peptide arising from mutant proteins in cancer cells or extracellular infected viruses that can bind to major histocompatibility complex (MHC) molecules. After the recognition by the MHC, antigens are brought to the surface of cells where they are identified by specific TCRs, which then initiates the downstream immune response. The binding between the peptides and MHC molecules plays an essential role in this complicated biological process.

**Fig. 1.**
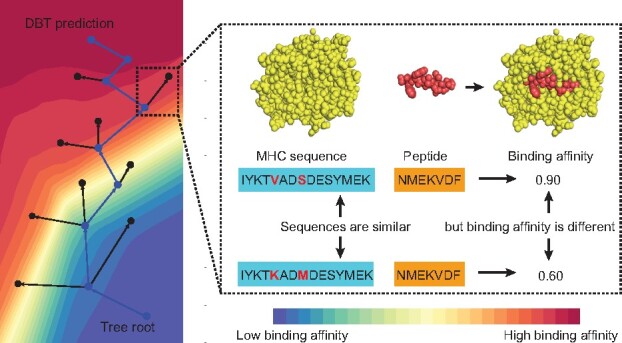
Overview of the DBTpred framework. DBTpred predicts the binding of peptides to MHC molecules by constructing a differential boundary tree

In the last decades, there have been lots of interests in developing computational methods that can accurately predict the binding affinity between peptides and MHC molecules. These methods can be mainly divided into two types: allele-specific and pan-allele approaches. The allele-specific models usually take only peptide sequences as input to train one model for each allele (Andreatta and Nielsen, 2016; Han and Kim, 2017; Nielsen and Andreatta, 2016), while pan-specific models take both peptide sequences and MHC pseudo-sequences as input to train a single model for all alleles (Han and Kim, 2017; Hoof *et al.*, 2009; Hu *et al.*, 2019; Jurtz *et al.*, 2017; Karosiene *et al.*, 2012; Liu *et al.*, 2017; Nielsen and Andreatta, 2016; 2017). Recently, deep neural networks (DNNs) have been proved to be powerful models and demonstrated the state-of-the-art performance in pan-specific tasks. For instance, NetMHCpan uses only a single layer neural network to predict the binding affinity between peptides and MHC molecules and achieves high prediction accuracy on both peptide-MHC binding affinity data and mass spectrometry data (Jurtz *et al.*, 2017; Nielsen and Andreatta, 2016). More carefully designed and precise neural networks have also been developed to address this problem. ConvMHC uses convolutional neural networks as a feature extractor of input sequences (Han and Kim, 2017) that can reliably predict the peptide binding of most HLA-A and -B alleles. ACME (Attention-based convolutional neural network for MHC epitope binding prediction) provides a novel sequence encoding method with a sophisticated network architecture to achieve a higher prediction accuracy (Hu *et al.*, 2019). It uses a head-to-head and tail-to-tail sequence encoding method and concatenates the output of the intermediate layer as the input to the fully-connected layer before the final output, which thus enables the information flow from the shallow layer to the deep (Hu *et al.*, 2019). These methods have been widely used in the discovery of tumor neoantigens to narrow down the lists of epitopes by providing high-quality rankings for candidates. However, they suffer from a high false-positive rate of predicted epitopes (“The Problem with Neoantigen Prediction”, 2017). In addition, these DNN based methods are biased toward learning the most determinant sequence motifs by smoothing the neighborhood signals and often fail in identifying mutation-induced tumor neoantigens since these neoantigens typically have only one single amino acid mutations that may significantly influence the MHC-peptide binding (Jiang *et al.*, 2019). Another limitation of these DNN models is their model interpretation. Most of these convolutional neural network based models provide multiple sequence motifs represented by a position-specific scoring matrix as model interpretation through examining the model weights of the convolutional layers (Han and Kim, 2017; Hu *et al.*, 2019). This type of interpretation is able to propose the informative regions on the protein sequences when the algorithm takes complete protein sequences as input. However, experimental results now have already identified a significant amount of residues whose mutations play essential roles in the immune response (Castle *et al.*, 2019). Therefore, more important biological knowledge in comparison to simple sequence motifs can help understand the logic of how the machine learning models make predictions based on these key residues and how these key residues interact with each other to determine the MHC-peptide affinity. In this work, we develop a new machine learning model, named DBTpred (Fig. 1), based on a differential boundary tree (DBT) (Zoran *et al.*, 2017), which can predict MHC-peptide binding affinity and at the same time provide a relatively transparent decision process as the model interpretation. The central idea of DBT is to organize all the training samples as a tree based on a distance metric calculated by a neural network model. For two connecting nodes (training samples) in the DBT, we require that their features are similar but their labels have to be significantly different. The prediction process is simply to search for the nearest neighbors along the tree from the root to the leaves. The intuition of this process is that we keep crossing the decision boundaries of the machine learning model to search for the nearest neighbor of the query sample. This model is specially designed to address the MHC-peptide binding problem in which proteins with similar sequences might have different binding affinities.

**Algorithm 1: Building the Boundary tree**

 /* ϵ determines the difference of labels */

 /* k is the maximum number of children per node */

 /* c(v) represents the label of node v */

 **1 Function** Building BT(D):

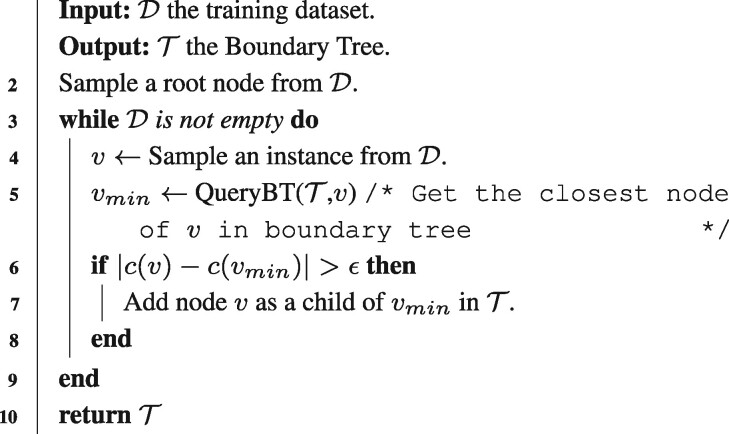

 11 **End Function**

To build the DBT model, we jointly construct the DBT structure and train the weights of the neural network. The major technical challenge is that the DBT structure search problem is a computationally demanding optimization problem which significantly limits both the scalability and training/test speed of the neural network model. To address this problem, we develop a new parallel algorithm to simultaneously train a deep neural network and construct a DBT by transforming the entire tree structure search process into multiple matrix operations. We demonstrated that our model achieved a superior performance in comparison to the state of art methods. We evaluated our model on the IEDB MHC class I binding affinity benchmark datasets (Kim, 2014; Vita *et al.*, 2015) and found that DBTpred can achieve better performance compared to the state-of-the-art MHC-peptide binding prediction methods. In addition, DBTpred can demonstrate the inner working logic to generate the final prediction, which enables us to identify important mutations that significantly alter the binding affinity.

## 2 Materials and methods

### 2.1 Boundary tree

Boundary tree (BT) is a special type of k-nearest neighbor (KNN) model, which was first proposed by Mathy *et al.* (2015). Compared to conventional KNN based models, BT has very appealing properties in reducing the computational and memory requirements of KNN methods. More importantly, BT can generate a decision path for the final prediction, which thus provides valuable insights to understand the logic of the model prediction. A BT model selects a subset of data instances to represent the whole training dataset by organizing these instances into a tree. To construct the tree structure, the algorithm randomly samples a data instance as the root of BT and then iterates all the training instances to search for the nearest neighbor by traversing the entire tree from the root based on a particular distance metric. At each step, if the label of the nearest neighbor is different from the query node (label difference >ϵ for a regression problem), the query node is added as the child of the current node of the tree. Thus, for two connecting nodes in a BT, their features should be similar while their labels are distinct. The algorithm details of building a BT is shown in Algorithm 1. To make a prediction for a query (test sample), if the query is much closer to the current node than any of its children, we assign the current node as the closest node and then transfer its label as the final prediction. Otherwise, the current node is updated to the child node that is closest to the query node, and then this process continues until reaching the leaf. The purpose is to search for the nearest neighbor but the search must follow the path of the tree from the root to leaves. The algorithmic details of querying a BT are shown in Algorithm 2.

**Algorithm 2: Querying the Boundary tree**

 /* ϵ determines the difference of labels */

 /* child(v) ={Child node set of v} */

 **1 Function** QueryBT (T,q) :

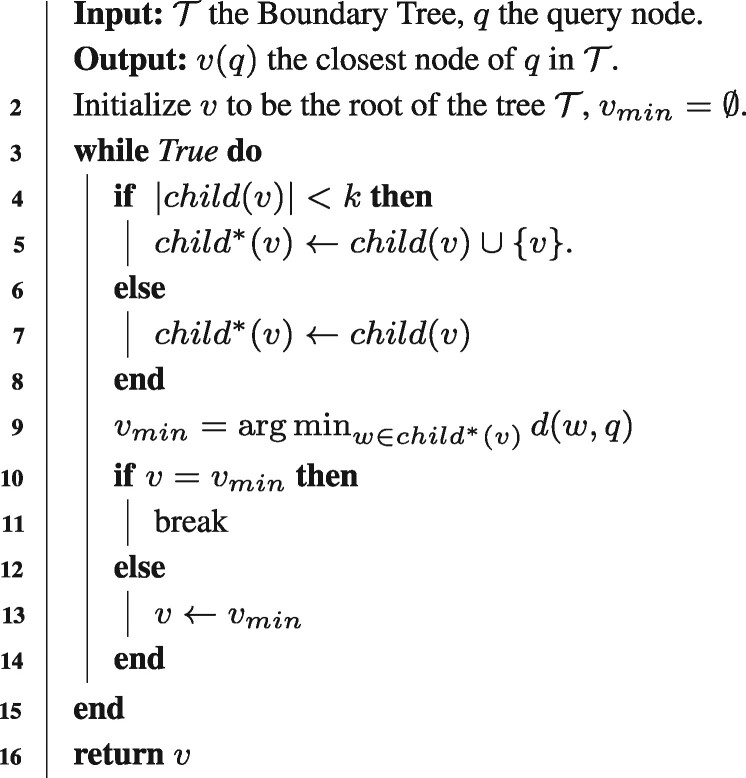

### 2.2 Differential boundary tree

Usually, BT requires an accurate metric to quantify the distance between training samples. To achieve this goal, Zoran *et al.* (2017) developed a new computational framework, named Differential Boundary Tree (DBT), which includes a deep neural network (DNN) to represent the node features in a low compact dimensional space. In particular, DBT implements a differentiable cost function enabling the simultaneous construction of the boundary tree and the weight optimization of the DNN model. In this work, we modify the training process of DBT to a relatively much simpler process. In particular, at each training epoch, we construct a temporal boundary tree on a batch of random sampled data points and then measure the loss function between the ground truth labels and their predictions from the temporal boundary tree. Another non-overlapping batch of training examples are sampled and then queried on the temporal boundary tree. Unlike the training algorithm proposed in Zoran *et al.* (2017) which maximized the log-likelihood of all the data collected from the transition paths, here we adopt the weighted summation of the labels of the nearest neighbor and its siblings on the temporal boundary tree as the final prediction of the query. Specifically, let $x^{*}$ stand for the nearest neighbor of query node *x*. Then the final prediction of *x* is

(1) $$y=\sum_{x_{i}\in s(x^{*})}\frac{\;\text{exp}(-d(f_{\theta }(x_{i}),f_{\theta }(x)))}{\sum_{x_{i}\in s(x^{*})}\;\text{exp}\;(-d(f_{\theta }(x_{i}),f_{\theta }(x)))}y_{i},$$

where *d* represents the Euclidean distance function, $s(x^{*})$ denotes the sibling nodes of $x^{*}$ and $x^{*}$ itself, *y_i_* stands for the label of *x_i_* and $f_{\theta }$ stands for the neural network with weights *θ*. Then, the loss function that we try to minimize is the mean square error loss defined as follows,

(2) $$L=\frac{1}{N}\sum_{y\in D_{\mathrm{query}}}{\left(y-\hat{y}\right)}^{2},$$

where *N* stands for the number of nodes in set $D_{\mathrm{query}}$, and $\hat{y}$ represents the label of *y*. We optimize the parameter in the neural network using the Adam algorithm (Kim, 2014) in each epoch. In the conventional error backpropagation training algorithm, the forward function makes the prediction and the backward function propagates the error to calculate the gradient for each weight. Here, DBT simply replaces the prediction from a forward function based on the prediction from a boundary tree. The algorithm details are demonstrated in Algorithm 3. When Algorithm 3 converges, we re-run Algorithm 1 on all the training samples to create the final DBT which is then used for making final prediction.

**Algorithm 3: Training the differential boundary tree (DBT)**

 /* D is the training dataset */

 /* T is the number of iterations */

 **1** Initialize the neural network $f_{\theta }$ with random weights.

 **2 while** *not converged* **do**

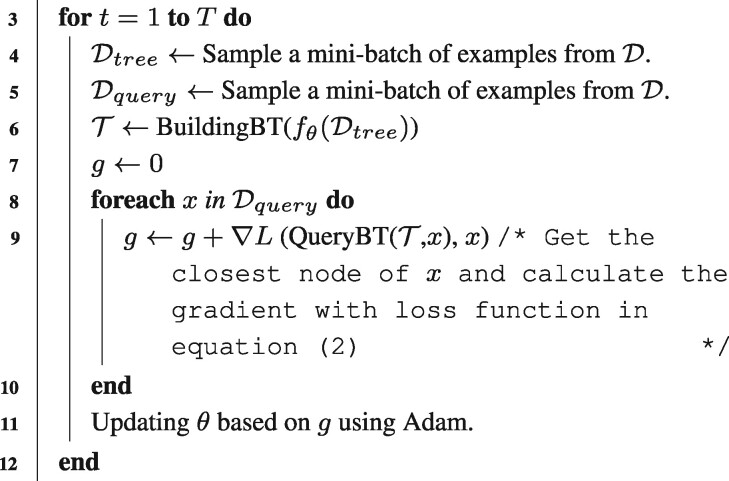

 **13 end**

### 2.3 A parallel DBT training algorithm

The major problem of DBT and BT is that their training process is generally quite slow. Training one model for a MHC-peptide binding prediction task with 10 000 samples needs about an hour. The training process is slow since it both involves a series of node querying operations on the temporal boundary trees and can only update the model parameters after collecting a number of traverse paths for all the samples in a sequential way, which cannot benefit from the modern parallelization frameworks such as Math Kernel Library (MKL) (Wang *et al.*, 2014) and Graphics Processing Units (GPUs).

**Fig. 2.**
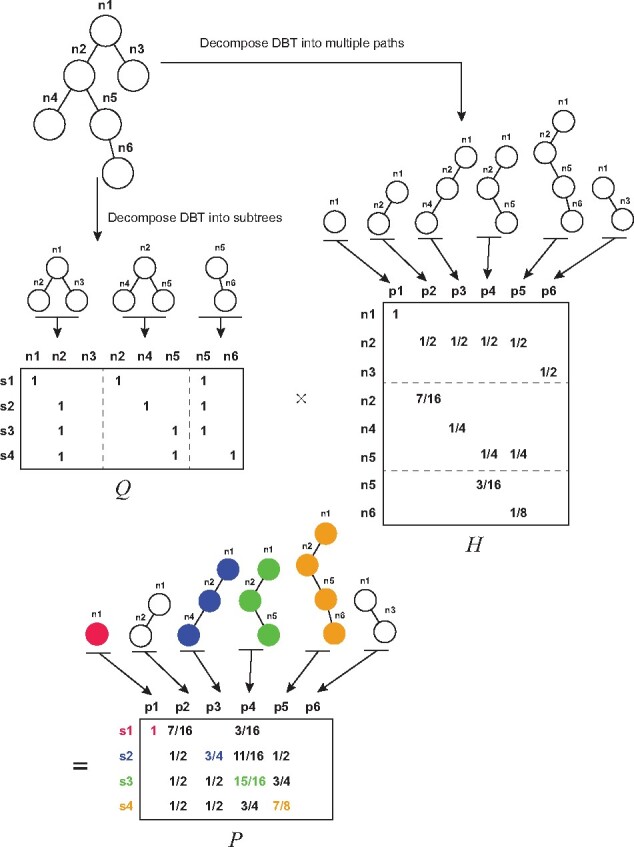
The illustration of a parallel algorithm for querying boundary trees

In this work, we also develop a new parallel training/test algorithm to accelerate this query process by transferring the path search process into a matrix multiplication problem, which can be easily parallelized through using the GPUs. First, we decompose the intermediate nodes of the boundary tree into different subtrees where each subtree contains one intermediate node and all of its children. Then we construct a query matrix *Q* in which rows represent query samples within the same batch and columns represent all the subtrees from the decomposition. For each query node, we searched for its closest neighbor in each subtree based on the Euclidean distance of neural network outputs. The closest neighbor is labeled as 1 and otherwise as 0 in the matrix *Q*. For instance, Suppose that the closest nodes of *s*_2_ in the three subtrees are *n*_2_, *n*_4_, *n*_5_, respectively, then the values corresponding to the *n*_2_, *n*_4_, *n*_5_ columns are labeled as 1 in row *s*_2_ (Fig. 2, left). Note that each subtree is independent of each other and this construction can be fully parallelized on different computational resources. Next, we construct a traverse matrix *H* in which rows represent subtrees and columns represent all the paths of boundary trees from the root (Fig. 2, right). For each edge (e.g. *n*_1_ to *n*_2_) in each path (column *p*_3_), we assign a score $\alpha^{d-1}$ to it, where *d* is the depth of the reachable node (*n*_2_) of the edge and *α* is a constant between 0 and 1 (which is set to 0.5 in practice). The score for a path is equal to the summation of all the scores on its edges. Note that the same edge can be assigned with different scores as it can belong to different paths. For a path stopping at non-leaf nodes, its score is equal to the mean of $\alpha^{d-1}$ and $\sum_{i=d}^{M-1}\alpha^{i}$, that is,

(3) $$\frac{1}{2}(\alpha^{d-1}+\sum_{i=d}^{M-1}\alpha^{i}),$$

where *M* stands for the maximum depth of the tree (which is 4 in Fig. 2). Note that this construction can be easily implemented by the matrix multiplication using the feature matrix of query and tree nodes. Then for each query sample *s_i_*, and corresponding row *Q_i_* and each column *H_j_*, we select the path of *s_i_* based on the following criterion,

(4) $$\mathrm{path}(s_{i})=\arg\max_{j}(Q_{i}^{T}H_{j})$$

It is easy to see that for a query sample *s_i_*, the inner product between *Q_i_* and *H_j_* results in the sum over all the edges scores of *s_i_* on the path *j*. Given the above definition, we introduce the following main theorem about our fast training algorithm, below

Theorem 1. *The optimal path found by Algorithm 2 is the same as that derived from Equation 4, when* α<1.

Proof 1. *To prove this theorem, we first define some notation. Let S(n) represent the assigned score for a reachable node n, and S(P) represent the total score of a path P. Then for an arbitrary path $P=(n_{1},\ldots ,n_{m})$, where n_i_ is the i-th node on the path, then total score of path P is $S(P)=\sum_{i=2}S(n_{i})$. We first prove the following lemma.*

Lemma 1. $S(n_{i})>S(P_{\mathrm{parent}}^{*}(n_{i}))$, where $P_{\mathrm{parent}}^{*}(n_{i})$ represents the paths that start from the parent of *n_i_* but do not pass *n_i_*. That is, the assigned score of node *n_i_* is larger than those of paths starting from the parent of *n_i_* but not passing through *n_i_*.

As shown in Figure 2, if a query node passes an edge $e=(n_{i-1},n_{i})$ in a subtree, the scores of $S(P_{\mathrm{parent}}^{*}(n_{i}))$ can be divided into following cases:

1. For a path that ends at a leaf node, the maximum score it can get is $\sum_{i=d}^{M-1}\alpha^{i}$, where *d* is the depth of node *n_i_*, and *M* is the maximum depth of the tree.
2. For a path that ends at a child node of the node $n_{i-1}$, the maximum score is $\frac{1}{2}(\alpha^{d-1}+\sum_{i=d}^{M-1}\alpha^{i})$, where *d* is the depth of node *n_i_*, and *M* is the maximum depth of the tree.
3. For a path that ends at a non-leaf node which is not a child node of the node $n_{i-1}$, the maximum score is $\sum_{i=d}^{g-1}\alpha^{i}+\frac{1}{2}(\alpha^{g-1}+\sum_{i=g}^{M-1}\alpha^{i})$, where *g* is the depth of a non-leaf node that the path stops, *d* is the depth of node *n_i_* and *M* is the maximum depth of the tree.

Considering all these three conditions, the maximum value of $S(P_{\mathrm{parent}}^{*}(n_{i}))$ is $\sum_{i=d}^{g-1}\alpha^{i}+\frac{1}{2}(\alpha^{g-1}+\sum_{i=g}^{M-1}\alpha^{i})$. Meanwhile, it is easy to see that $S(n_{i})=\alpha^{d-1}$. Then we can see that the maximum of $S(P_{\mathrm{parent}}^{*}(n_{i}))$ is smaller than $S(n_{i})=\alpha^{d-1}$ since,

(5) $$\alpha^{d-1}\geq \sum_{i=d}^{g-1}\alpha^{i}+\alpha^{g-1}>\sum_{i=d}^{g-1}\alpha^{i}+\frac{1}{2}(\alpha^{g-1}+\sum_{i=g}^{M-1}\alpha^{i})$$

This equation holds when d≥2 and g≥3 which are easily satisfied in real world applications. Therefore, Lemma 1 has been proved.

Finally, from Lemma 1 we have $S(P)=\sum_{i=2}S(n_{i})>\sum_{i=2}S(P_{\mathrm{parent}}^{*}(n_{i}))$, which means that at each node $n_{i}(i\geq 2)$ of the path *P*, the score of any other branch paths that starts from $n_{i-1}$ and does not pass *n_i_* are smaller than *S*(*P*). Thus, Theorem 1 has been proved.

### 2.4 Architecture of deep neural network

**Fig. 3.**
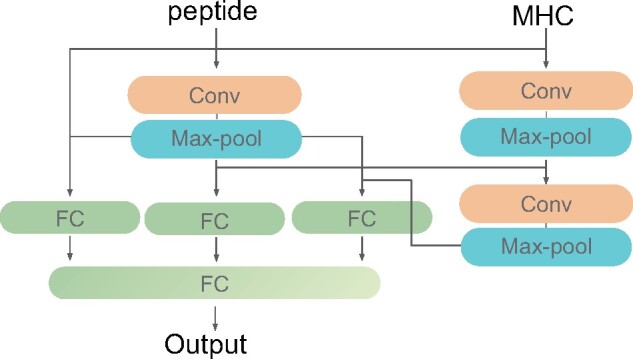
Neural network architecture of DBTpred. The input data first passes 1–2 convolutional layers (Conv) and then the outputs of two branches are concatenated as the input to the three fully-connected layers (FC). Finally, the outputs of fully-connected layers are concatenated and pass through another fully-connected layer to produce the final representation features

The peptides and MHC pseudo-sequences were first encoded as in Hu *et al.* (2019). As shown in Figure 3, the protein sequences were first encoded by a BLOSUM50 scoring matrix (Henikoff and Henikoff, 1992) into encoded vectors and then passed to 1–2 convolutional layers. In particular, we use one convolutional layer to model the peptide sequence and two convolutional layers to model the MHC protein sequences. The outputs of the two branches are then concatenated as the input to the three fully-connected layers. In the end, the outputs of fully-connected layers are concatenated and pass through another fully-connected layer to output the final representation features.

### 2.5 Saliency map scores

As a query node traverses along the boundary tree, it continually searches for the nearest tree node based on the distance metric derived from the feature representation of deep neural networks. Specifically, let $x\in R^{20\times L}$ be the one-hot encoded sequence of an arbitrary tree node on the transition path and $q\in R^{20\times L}$ be the one-hot encoded sequence of query node, where *L* is the length of sequence, we can obtain the distance between *x* and *q* as

(6) $$d(x,q)={\left(f_{\theta }(Bx)-f_{\theta }(Bq)\right)}^{2},$$

where *B* is the BLOSUM50 matrix, and $f_{\theta }$ is the deep neural network. To illustrate the important residues that significantly influence the distance *d*(*x*, *q*), we calculated the saliency map (Simonyan *et al*., 2013) of *x* and *q* with respect to *d*(*x*, *q*), that is,

(7) $$w_{x}=\frac{\partial d(x,q)}{\partial x},w_{q}=\frac{\partial d(x,q)}{\partial q}.$$

Then, we multiplied *w_x_* and *w_q_* by their one-hot encoded matrices *x* and *q*, respectively to get the derivatives of the actual residues of sequences, noted as $w_{x}^{*}$ and $w_{q}^{*}$, respectively. Finally, we normalized the saliency map by dividing its maximum absolute values, that is $\frac{w_{x}^{*}}{\max\left|w_{x}^{*}\right|}$ and $\frac{w_{q}^{*}}{\max\left|w_{q}^{*}\right|}$. Thus, the saliency map scores demonstrate the important residues between the distance of query and tree nodes along the traverse path in the boundary tree.

## 3 Results

### 3.1 Performance of DBTpred

To evaluate the performance of DBTpred, we conducted a five-fold cross-validation and compared DBTpred to the state-of-the-art method NetMHCpan 3.0 (Nielsen and Andreatta, 2016), which is a pan-specific method and also trained on the IEDB MHC class I binding affinity dataset (Kim, 2014; Vita *et al.*, 2015). NetMHCpan 3.0 is probably the most widely used method in the prediction of MHC-peptide binding affinity and also provides detailed performance for different alleles and peptide lengths. Therefore, we trained our DBTpred model on the same IEDB dataset and investigated the performance of our method on the same alleles and peptide lengths (i.e., 9-mer, 10-mer and 11-mer).

**Fig. 4.**
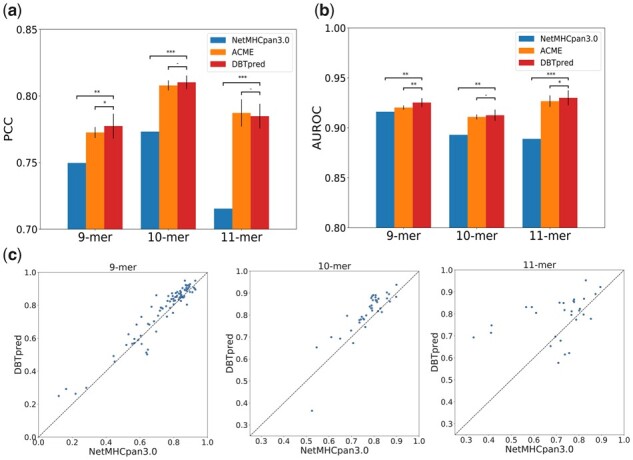
Performance comparison in five-fold cross-validation between DBTpred and different baselines in distinct peptide lengths in terms of (**a**) Pearson correlation coefficient (PCC), and (**b**) area under receiver operating characteristic curve (AUROC). (**c**) The performance comparison between DBTpred and NetMHCpan 3.0 for different alleles. *0.05<P<0.1, **0.001<P<0.05, ***P <* *0.001, ^-^*P *>* *0.1 (one-sample *t*-test based on the allele performance between DBTpred and NetMHCpan3.0 and Wilcoxon rank-sum test based on the allele performance between DBTpred and ACME)

The prediction performance is measured in terms of Pearson correlation coefficient (PCC). As shown in Figure 4a, DBTpred increased the PCC by 2.8%, 3.7% and 6.9% in comparison to NetMHCpan 3.0 for 9-mers, 10-mer and 11-mers, respectively. We also investigated the performance in terms of the area under receiver operating characteristic curve (AUROC). As shown in Figure 4b, DBTpred increased AUROC by 0.91%, 2.0% and 4.1% compared to NetMHCpan 3.0 for 9-mers, 10-mers and 11-mers, respectively. When investigating the performance of individual alleles in peptide lengths, we found that DBTpred outperformed NetMHCpan 3.0 in the vast majority of alleles. More specifically, for 88 alleles of 11-mers, the performance of DBTpred was larger than NetMHCpan 3.0 in 21 out of 29 alleles. Among them, 5 alleles achieved over 20% improvements in PCC. For 10-mers and 9-mers, the performance in 33 out of 40 alleles and 70 out of 88 alleles achieved performance improvement compared with NetMHCpan 3.0. For 9-mers, among 19 alleles DBTpred improved over 5% in PCC and for 10-mers, among 15 alleles DBTpred improved above 5% over the baseline (Fig. 4c).

To evaluate the contribution of DBT, we also compared the performance of DBTpred to ACME (Hu *et al.*, 2019), which used the same neural network architectures as in DBTpred. We found that DBTpred still achieved better performance compared with ACME for most of the cases although in 11-mer DBTpred decreased a little in terms of PCC (Fig. 4, Supplementary Fig. S1). These results revealed that DBTpred can accurately predict the binding affinity of peptides and MHC class I molecules.

**Fig. 5.**
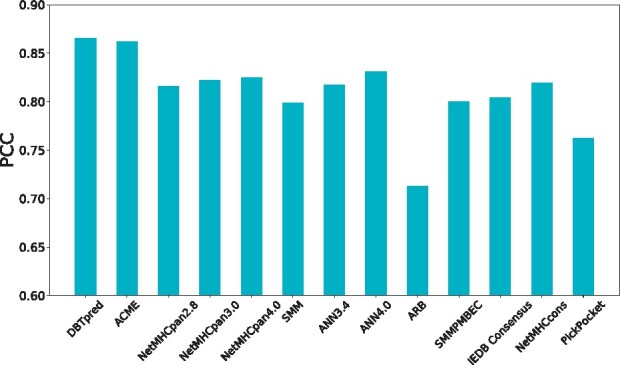
Performance comparison between DBTpred and other state-of-the-art methods obtained from website (http://tools.immuneepitope.org/auto_bench/mhci/weekly/) on an independent test dataset (Trolle *et al.*, 2015) in terms of Pearson correlation coefficient (PCC)

To further demonstrate the generalizability of DBTpred, we tested its performance on an independent MHC class I binding affinity benchmarking dataset (Trolle *et al.*, 2015), which contains over 30 000 MHC class I binding peptides. In our study, we tested the performance on the peptides with IC50 binding affinity measurement in the benchmarking dataset. We trained DBTpred on the training dataset and compared its performance to that of other state-of-the-art methods. The prediction results of these state-of-the-art methods were downloaded from the benchmarking website (http://tools.immuneepitope.org/auto_bench/mhci/weekly/). As shown in Figure 5, DBTpred outperformed all the other benchmarking methods. Compared to the NetMHCpan3.0, DBTpred achieved an increase of 4.3% in PCC. We also retrained the ACME (Hu *et al.*, 2019) with the same training dataset and compared its performance to that of DBTpred. We found that DBTpred still obtained better performance compared to ACME.

### 3.2 Training DBTpred

**Fig. 6.**
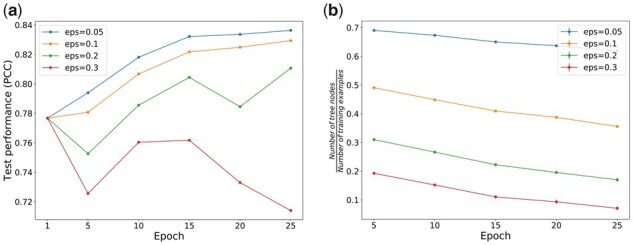
Performance of the training process. (**a**) The test performance of DBTpred vs. the number of training epochs. (**b**) The ratio of the number of tree nodes to the total number of training examples vs. the number of training epochs

During the training of DBTpred, the model iteratively built a boundary tree with a subset of training data, and traversed this tree using another subset of training data to update the parameters of the backbone neural network. When building the boundary tree, we ensured that the absolute difference between the query node and its closest node was larger than a threshold (denoted as ϵ), otherwise it was be discarded. This process is slow when trained on a large dataset (Zoran *et al.*, 2017). To address this problem, we first pre-trained a conventional neural network (only one epoch) and then used the weights except for the last output layer as the initiation to our backbone. Next, we used our parallel training algorithm to accelerate the query process of the boundary tree, which was the most time-consuming step in the whole training process. In our study, we found that a good initiation of backbone enabled the training process to converge fast. Figure 6a illustrates how the performance changed with the number of training epochs in DBTpred. We found that the label difference parameter should be larger than 0.1 to ensure a model with good training performance. When was too small, the performance of DBTpred fluctuated, since there were less samples in the boundary tree to represent the whole dataset.

Another observation was that the larger, the less number of nodes was left in the tree. As shown in Figure 6b, all the boundary trees with small contained more nodes than those with large. In addition, the ratio of the number of tree nodes to the total number of training samples decreased as the number of training epochs in DBTpred. This result indicated that the training process enabled the model to learn good feature representation such that the boundary tree can represent the whole dataset with less nodes. For example, after 25 training epochs, DBTpred only needed 40% training examples to achieve over 0.8 of test performance in terms of PCC.

**Fig. 7.**
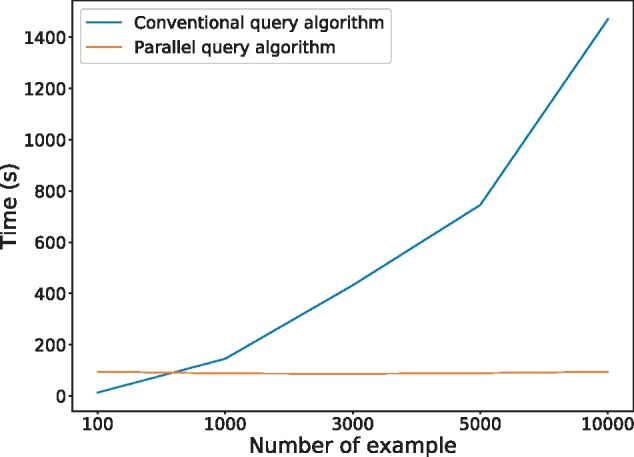
Comparison of computational time between the conventional query algorithm of boundary tree (Mathy *et al.*, 2015) and our parallel querying algorithm

In addition, our parallel algorithm transferred the discrete query process into matrix multiplications, which can be easily calculated on GPUs. As illustrated in Figure 7, compared to the original query algorithm (Mathy *et al.*, 2015), whose training time was linearly proportional to the number of samples, our method exhibited almost a constant growth rate, which thus can greatly speed up the training process.

### 3.3 Feature representation

**Fig. 8.**
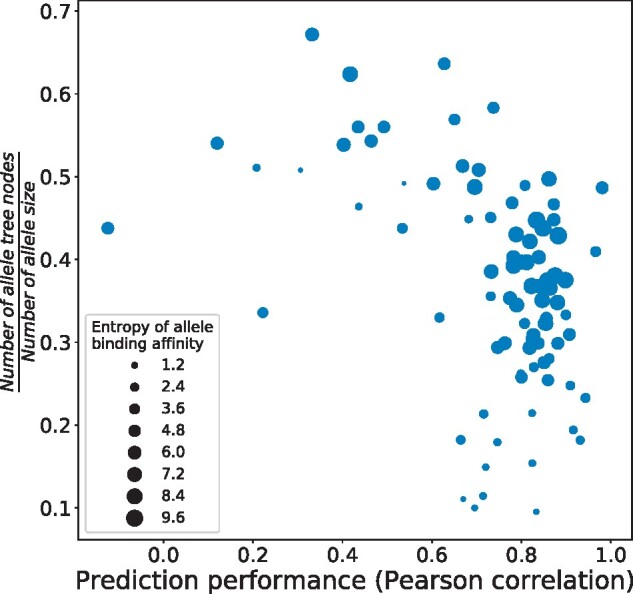
Correlation between the ratio of the number of allele tree nodes (allele samples left in boundary tree) to the total number of allele samples and the performance of individual alleles

We investigated how many samples are generally needed in DBTpred for each allele. Intriguingly, we found that for each allele, if there were more samples of the allele left in the boundary tree, the smaller was the performance of this allele. As shown in Figure 8, most alleles can be represented by only 30–40% of all the samples using the boundary tree and achieved over 0.7 of test performance, while some alleles needed to be represented by over 50% samples in the boundary tree and exhibited less than 0.5 of test performance. The sequence patterns of these alleles were not completely learned by the model since although they occupied large amounts of samples in the boundary tree, they did not achieve a good performance compared with those of other alleles. Notably, we also found that there were some alleles which left small amounts of samples in the boundary tree while still achieving good performance (e.g. alleles in bottom right corner in Fig. 8), which indicated that the features of these alleles may be easy to learn. Figure 8 also showed the label complexity of individual alleles (measured as the entropy of the allele labels). In fact, these alleles with less tree nodes while achieving high performance were those with low label complexity (e.g. most of the labels are 0). Thus, the boundary tree needed a very small amount of samples to represent the features of these alleles. In summary, the statistical properties of the boundary tree demonstrated how well the model was trained for the task. In some cases, the ratio of the number of samples left in trees to the total number of samples actually indicated the complexity of the learning.

### 3.4 Model interpretation

Most deep learning models are end-to-end methods which input test samples into a deep neural network and then output prediction results. Although deep learning has exhibited strong predictive power in lots of biological problems, researchers are interested in analyzing the interpretability of models which may provide useful biological understandings. There have been many developed methods in analyzing deep neural networks such as LIME (Ribeiro *et al.*, 2016), saliency map (Simonyan *et al.*, 2013), GradCam (Selvaraju *et al.*, 2017) and DeepLIFT Shrikumar *et al.* (2017). These methods are able to decipher which part of input plays an important role in the final prediction. However, deeply interpreting a conventional deep learning method is still a popular research direction (Lipton, 2018; Simonyan *et al.*, 2013).

**Fig. 9.**
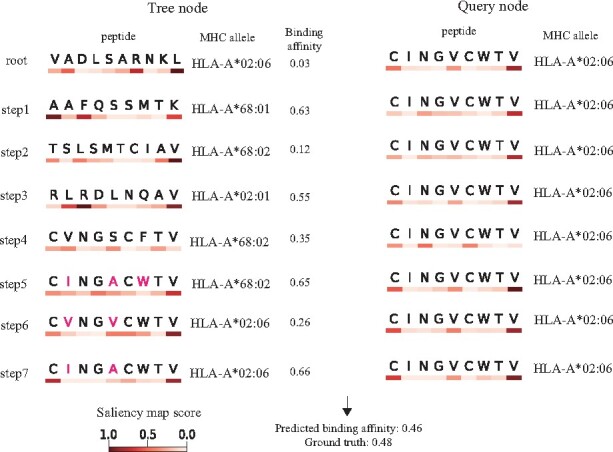
Illustration of a query path in DBTpred. The normalized saliency map scores are shown on the bottom

DBTpred takes advantage of the strong learning power of deep neural networks to learn a good representation of datasets, through organizing the datasets into a tree structure. Thus, when predicting a test sample, DBTpred can provide an interpretable decision path of the tree, which may provide useful interpretation. Figure 9 illustrates a simple example of the interpretation of how DBTpred predicted a test sample. First, we calculated the saliency map (Simonyan *et al.*, 2013) in terms of the Euclidean distance between the test sample and each tree node. The saliency map scores pointed out the important residues in determining the sample distance. DBTpred clearly demonstrated how the test sample traversed to its closest node step by step. In particular, the model first searched for the last valine in the peptide which occupied the largest saliency map score (step 1 to step 3), and then it searched for cysteine and two valines in the first, fifth and the last positions of peptide (step 3 to step 7). These three residues are the key residues in determining the prediction of binding affinity of the test sample. In addition, apart from these three residues, the path also displayed several possible point mutations which may strongly alter the binding affinity of the test sample that can be smoothed out by the deep neural network, e.g. the mutation between valine and isoleucine on the second residue of the peptide (marked as crimson in Fig. 9). Moreover, since the peptide sequences and MHC pseudo-sequences are both passed through the deep neural network, we can also calculate the saliency scores along the MHC sequence to demonstrate the important residues influencing the final prediction of binding affinities (Supplementary Fig. S2). In summary, DBTpred enabled an easy interpretation of how the model makes a prediction of peptide-MHC binding affinity and also provided possible mutation residues that may influence the binding affinity.

## 4 Discussion

In this article, we demonstrated that DBTpred can achieve an accurate prediction about MHC class I binding affinity. We developed a parallel training algorithm to accelerate the most time-consuming step of the differential boundary tree and made it possible to apply DBTpred into a large dataset. We also demonstrated that the traverse path of the test sample can provide an interpretable visualization of how the model predicted the final output. Meanwhile, DBTpred detected the possible residue mutations that can largely influence the binding affinity of the test sample which was missed by conventional deep neural networks. In addition, like other KNN based methods, DBTpred searches for the nearest training samples for queries and uses the weighted summation of the labels of the training examples and their children as the final prediction. This means that it is easy for DBTpred to evaluate the reliability of its prediction based on the distance between queries and their closest nodes (Supplementary Fig. S3). However, although we have vastly increased the training and inference speed of DBTpred, it was still quite slow compared to conventional neural networks. The training of DBTpred on the total dataset still requires about two days. When building the boundary tree, the training samples were randomly sampled from the training dataset, which may influence the structure of the boundary tree. Thus, the paths for the test samples may be different in the boundary trees. In practice, we built several boundary trees and query the test samples in these trees to choose a path with good interpretation. We also calculated the averaged cosine distance of the saliency map between the test sample and the traverse nodes as the interpretable score for the path. In summary, DBTpred provides a novel method that can accurately predict the MHC class I binding affinity and provide detect useful mutation residues affecting the binding affinity.

## Supplementary Material

btab312_Supplementary_Data
