# Supplementary material for: Predicting MHC-peptide binding affinity by differential boundary tree
Source: Bioinformatics. 2021 Jul 12;37(Suppl 1):i254–61. doi: 10.1093/bioinformatics/btab312 (PMC8275335; doi:10.1093/bioinformatics/btab312)
Supplement: btab312_Supplementary_Data [file btab312_supplementary_data.pdf]

Supplementary Material for *Predicting MHC-peptide binding affinity by differential boundary tree*

Peiyuan Feng [1], Jianyang Zeng [1,2,*], Jianzhu Ma [3,4,*]

[1]Institute for Interdisciplinary Information Sciences, Tsinghua University, Beijing, China.
[2]MOE Key Laboratory of Bioinformatics, Tsinghua University, Beijing, China.
[3]Department of Computer Science, Purdue University, West Lafayette, IN 47907, USA.
[4]Department of Biochemistry, Purdue University, West Lafayette, IN 47907, USA.

Figures

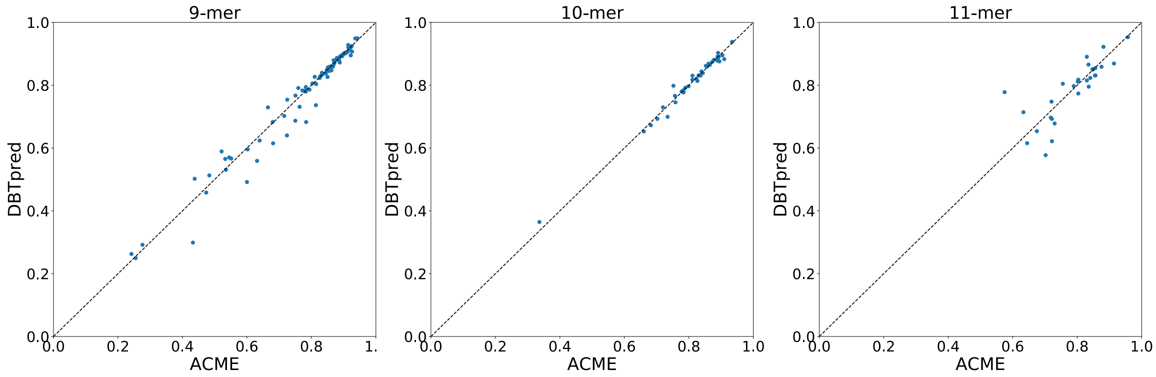

Fig. S1. Performance comparison between DBTpred and ACME for different alleles.

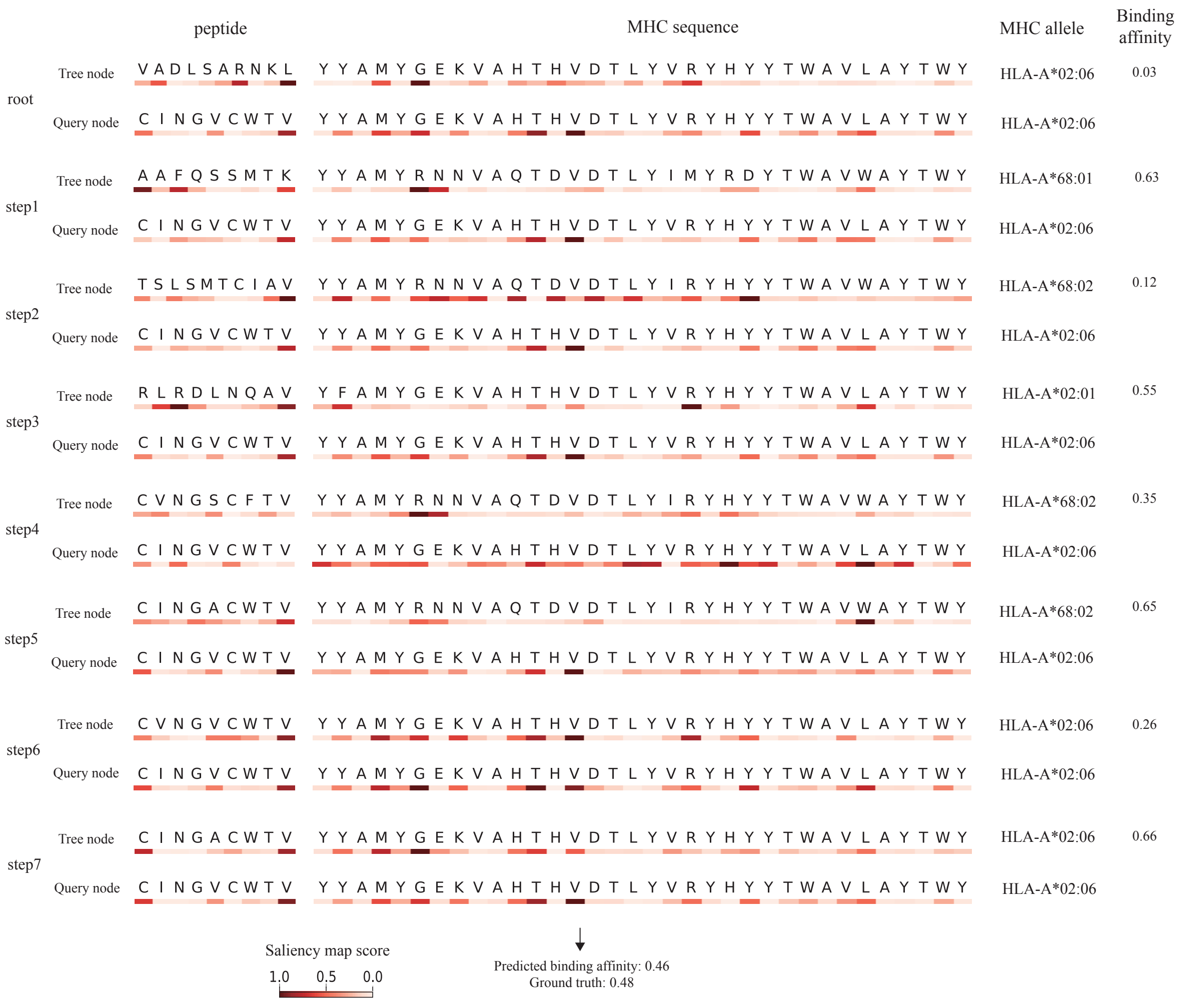

Fig. S2. Comprehensive illustration of query path in DBTpred. The normalized saliency map scores are shown on the bottom.

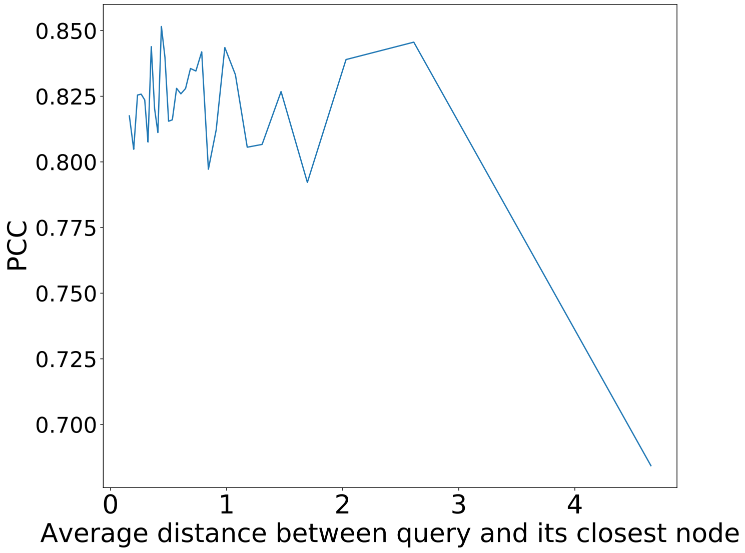

Fig. S3. Relations between query node distance to its closest node and prediction performance. The prediction performance remains stable until the distance is larger than 3, after which the performance decreases fast.
